# Characterization of feed efficiency-related key signatures molecular in different cattle breeds

**DOI:** 10.1371/journal.pone.0289939

**Published:** 2023-09-27

**Authors:** Chaoyun Yang, Zengwen Huang, Cuili Pan, Shuzhe Wang

**Affiliations:** 1 College of Animal Science, Xichang University, Xichang City, Sichuan Province, China; 2 Key Laboratory of Ruminant Molecular and Cellular Breeding, School of Agriculture, Ningxia University, Yinchuan City, Ningxia, China; Government College University Faisalabad, PAKISTAN

## Abstract

Feed efficiency is a major constraint in the beef industry and has a significant negative correlation with residual feed intake (RFI). RFI is widely used as a measure of feed efficiency in beef cattle and is independent of economic traits such as body weight and average daily gain. However, key traits with commonality or specificity among beef cattle breeds at the same level of RFI have not been reported. Accordingly, the present study hypothesized that signatures associated with feed efficiency would have commonality or specificity in the liver of cattle breeds at the same RFI level. By comparing and integrating liver transcriptome data, we investigated the critical signatures closely associated with RFI in beef cattle using weighted co-expression network analysis, consensus module analysis, functional enrichment analysis and protein network interaction analysis. The results showed that the consensus modules in Angus and Charolais cattle were negatively correlated, and four (turquoise, red, tan, yellow) were significantly positively correlated in Angus liver, while (turquoise, red) were significantly negatively correlated in Charolais liver. These consensus modules were found to be primarily involved in biological processes such as substance metabolism, energy metabolism and gene transcription, which may be one of the possible explanations for the difference in feed efficiency between the two beef breeds. This research also identified five key candidate genes, *PLA2G12B*, *LCAT*, *MTTP*, *LCAT*, *ABCA1* and *FADS1*, which are closely associated with hepatic lipid metabolism. The present study has identified some modules, genes and pathways that may be the major contributors to the variation in feed efficiency among different cattle breeds, providing a new perspective on the molecular mechanisms of feed efficiency in beef cattle and a research basis for investigating molecular markers associated with feed efficiency in beef cattle.

## Introduction

Cattle breeds and feed efficiency (FE) are the most important factors in beef production. Breeds determine to a large extent the grade and quality of beef, with Japanese Wagyu and Angus cattle being recognized worldwide as breeds that produce higher quality meat (marbling score), while breeds such as Simmental and Charolais cattle provide higher beef production. Meanwhile, FE is also having a profound impact on the development of the beef industry. Residual feed intake (RFI) is the most commonly used measure for the assessment of FE in dairy cattle [1] and beef cattle [2]. RFI shows a moderately strong correlation with FE [3] and has no correlation with economic traits such as average daily gain, growth rate and fat depth [3–7], indicating that it can be used to breed high-FE beef herds with a balance of growth and economic traits. Meanwhile, RFI serves as a moderate heritability parameter [8–10], allowing consistent genetic gains to be achieved when used as part of a selection index. RFI levels vary with several factors, such as protein turnover, tissue metabolism, stress, digestibility, heating increment, fermentation, physical activity, body composition and feeding pattern [11]. However, the availability of signaling molecules closely related to RFI in cattle breeds has not been reported.

Potential mechanisms of phenotypic regulation by specific genes can be revealed using transcriptome sequencing technologies (such as KLF6 [12,13]), but most studies of RFI have focused on gene expression in one or two tissues of a breed [2,14–18]. In different studies, differences in experimental conditions (e.g. breed, age, feeding practices) often also lead to the differences in gene expression [13,19], making the search for RFI-related molecular markers a hot research topic. Therefore, more attention should be paid to searching for RFI-related signatures. It is well known that the liver is a major metabolic and energy producing organ. It is involved in many biological processes in the body and performs many essential functions such as detoxification and energy supply [20]. In studies on RFI in the liver of beef cattle, it was found that low RFI in the liver mainly elevated the efficiency of substance metabolism and energy metabolism, and reduced the energy required for biological processes such as immunity and inflammation [19]. At the same time, even at the same level of RFI, gene expression varies dramatically between studies [13]. Therefore, discovering the possible commonality of RFI between breeds using some specific bioinformatics methods is the way to uncover RFI variation.

Based on the hypothesis that RFI may have common signaling molecules in bovine liver of different breeds, the present study aims to identify key molecules regulating RFI in beef cattle. Consensus module analysis based on weighted co-expression network analysis (WGCNA) (which is a tool used to discover genes associated with complex phenotypic traits and has been used to discover the metabolites and signatures of genes associated with complex phenotypic traits [21,22]) was used to reveal co-expression modules and relevant gene clusters closely related to RFI, providing a new insight and horizon for the selection and identification of beef cattle breeds with high feed efficiency.

## Materials & methods

**Fig 1:**
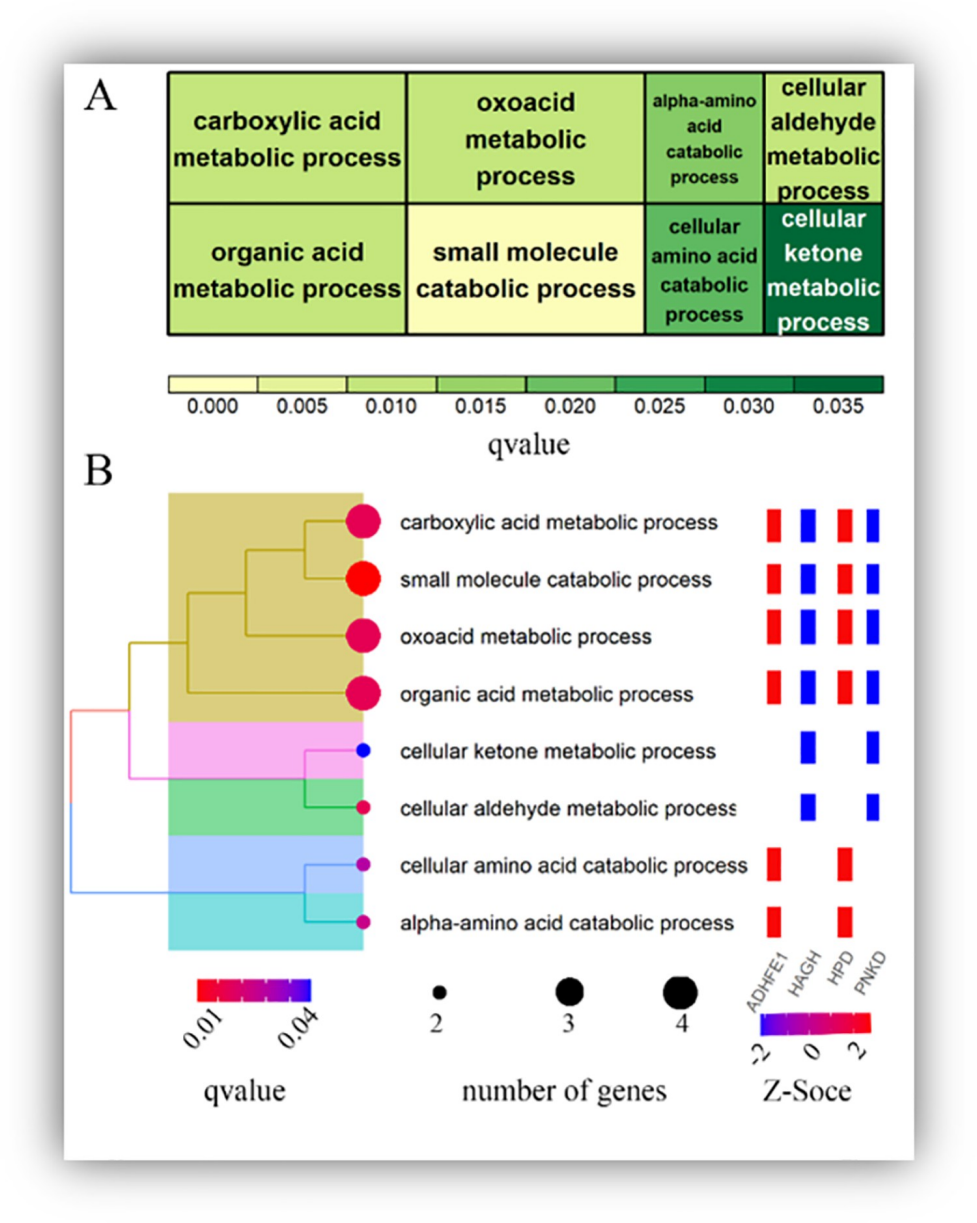
The data analysis process.

The data analysis process for the text is shown in Fig 1, with detailed steps in the detailed steps of the methodology that follow.

### Data preparation and processing

In this study, the GSE116775 dataset [23] was downloaded from the Gene Expression Omnibus (https://www.ncbi.nlm.nih.gov/geo/query/acc.cgi?acc=GSE116775). This project included four tissues (rumen, liver, fat, and muscle) from three beef breeds (Kinsella, Angus, and Charolais) with diverse **RFI** (sequencing platform: Illumina HiSeq 4000; Library layout: pair-end; Library source: TRANSCRIPTOMIC; Average length: 200), including eight animals of each breed with high or low **FE**. In the current study, transcriptome data from liver tissues of Charolais and Angus cattle were used for further analysis to explore consensus network modules and key gene clusters under identical feed efficiency conditions in the livers of different beef cattle breeds. After the original file (*fastq* format) was downloaded, the fastqc software [24] (version 0.11.7, https://www.bioinformatics.babraham.ac.uk/projects/fastqc/) and Trim-galore (version 0.6.6, https://www.bioinformatics.babraham.ac.uk/projects/trim_galore/**, parameters**: paired,—quality 25—length 36, stringency 3) were used to control the quality of reads, respectively. The quality-controlled clean reads were then aligned (**hisat2**, version 2.2.1, http://daehwankimlab.github.io/hisat2/; **parameters**: default parameters) to the index file of bovine reference genome ARS-UCD1.2 (downloaded from the *BovineGenome*.*org* website, https://bovinegenome.elsiklab.missouri.edu/downloads/ARS-UCD1.2) to obtain a *sam* file. The *sam* files were converted to the *bam* files using samtools software [25] (version 1.9, https://sourceforge.net/projects/samtools/files/samtools/1.9/; **parameters**: -b -S -h), and the *bam* file index was constructed, following gene quantification using the featureCounts program in the subread package [26] (version 2.0.1, http://subread.sourceforge.net/; **parameters**: -t exon -g gene_id) to acquire the count matrix, and the TPM value (Transcripts Per Kilobase of exon model per Million mapped reads) was selected to show the level of gene expression.

The median absolute deviation (MAD) was used to remove genes with abnormal TPM values as input to the WGCNA [27]. First, the MAD value of all genes was calculated; then, all genes in the first quartile (Q1) were retained, and finally, genes with a MAD greater than one were retained. The overlap in the Angus and Charolais liver was used to construct the consensus module.

### Network construction and module detection

The WGCNA methodology was used to construct the network and identify the consensus module. Before proceeding with network construction, sample outliers were checked. The Euclidean distance between samples was calculated using the hclust function in the WGCNA package, with the parameter method = "average", and samples with clear outliers were removed. The construction of a weighted gene network requires the optimal selection of the soft threshold power β, which improves the co-expression similarity and calculates the adjacency. Therefore, the selection of the optimal soft threshold power β was performed using the function pickSoftThreshold (based on the approximate scale-free topology criterion) in the R package WGCNA [28].

After eliminating the outliers and obtaining the optimal soft thresholding power β, the blockwiseConsensusModules function was used to compute the topological overlap of the co-expression network and generate the module. Here we used power = soft threshold power β (when R = 0.85). The number 30 was used as the minimum number (minModuleSize = 30), the module detection sensitivity was 2 (deepSplit = 2), and the cut height for merging modules was 0.25 (mergeCutHeight = 0.25, i.e., merge into one module if the correlation coefficient of eigengenes within the module is more significant than 0.75). To avoid rearrangement of eigengenes within modules according to KME, the parameter minKMEtoStay was set to 0 and the parameter maxBlockSize was set to 5000, and the remaining parameters were set as default. When the RFI-related modules in Angus cattle were obtained, the hypergeometric test (Fisher’s exact test) was used to check for overlap.

The consensus module was then analysed with respect to the phenotypic RFI. First, the co-expression networks for each object used in the consensus network were associated with the RFI. The species-specific co-expression network was built using the blockwiseModules function with the following parameters: power = soft threshold power β. TOMType = "unsigned", the minModuleSize = 30. mergeCutHeight = 0.25. maxBlockSize = 20000. pamRespectsDendro = FALSE, verbose = 3, other parameters were set as default. This process generates species-specific co-expression modules associated with phenotypic RFI (significant correlations). If the correlation coefficients used for the species-specific and RFI had the same sign (zero relationships when the two correlations have opposite signs, labelled "NA"), the minimum correlation coefficient and the maximum significance test p-value were retained to evaluate the relationship between the two modules. This procedure makes it possible to unify the common features and similarities between the two modules.

Finally, the gene significances (GS) and module memberships (MM, also known as KME) of the eigengenes in the species-specific consensus module were calculated using the corAndPvalue function. To determine the relationship between the two species-specific co-expression modules, a "meta-analysis" was performed to determine their correlations. Once the consensus network modules were obtained, the genes within the significantly related modules of the consensus or species-specific network were subjected to functional enrichment analysis to elucidate the biological processes and signaling pathways, and protein-protein interaction (PPI) analysis to discover the core genes and key regulatory subnetworks (if the number of genes in the module was more than 300, the top 300 absolute values of GS were selected for subsequent analysis).

### PPI and key gene analysis

Once the consensus module of interest is obtained, the relationship of genes within the module is explored using protein network interaction analysis. Protein network interactions were obtained using the Strings website (https://string-db.org/, version 11.0) with the following parameters: Organism: Bos taurus; the minimum required interaction score was set to high confidence (0.7), other parameters were set to default to obtain high confidence protein network interactions. The CytoHubba plugin in Cytoscape was used to detect hub genes by four centrality methods, which were network topology analysis—degree, edge percolated component (EPC), maximum clique centrality (MCC) and maximum neighbourhood component (MNC), which are practical methods for identifying hub genes from PPI networks [29]. The overlaps of the four methods (the highest top20) were defined as hub genes. The MCODE plugin was applied to identify critical sub-networks and the seeds of nodes (the seeds of nodes were also defined as hub genes), and the parameter configuration is degree cutoff = 2, node score cutoff = 0.2, k-core = 2, and maximum depth = 100. Subsequently, genes from key subnetworks were subjected to functional enrichment analysis.

### Gene function classification and annotation

The R package clusterProfiler (version 4.05) was used to annotate and visualize gene functions. The enrichGO function is applied to the Gene Ontology annotation, which includes a biological process (BP), a molecular function (MF) and a cellular component (CC), with the following parameters: pvalueCutoff = 0.05 (adjusted P-value cutoff for enrichment tests), qvalueCutoff = 0.2 (q-value cutoff for enrichment tests), pAdjustMethod = "BH" (multiple test correction method for p-values, i.e, Benjamini & Hochberg method), and the maximum number of genes enriched in the pathway maxGSSize and the minimum number minGSSize are adjusted according to the size of the annotated gene set. The enrichKEGG function was adapted to the Kyoto Encyclopedia of Genes and Genomes (KEGG) annotation to reveal the relevant pathways with the same parameters as the enrichGO function. All enrichment analysis results were visualised using the R package ggplot2.

## Results

### Consensus module detection

Using the MAD method, we finally determined that the number of genes fulfilling the conditions in Angus and Charolais livers was 8,547 (S1 Table) and 8,484 (S2 Table), respectively. Genes co-expressed in both Angus and Charolais livers (7,534) were used to construct the consensus module, and the remainder were defined as genes specifically expressed in Angus (1,013) or Charolais (950) livers.

**Fig 2:**
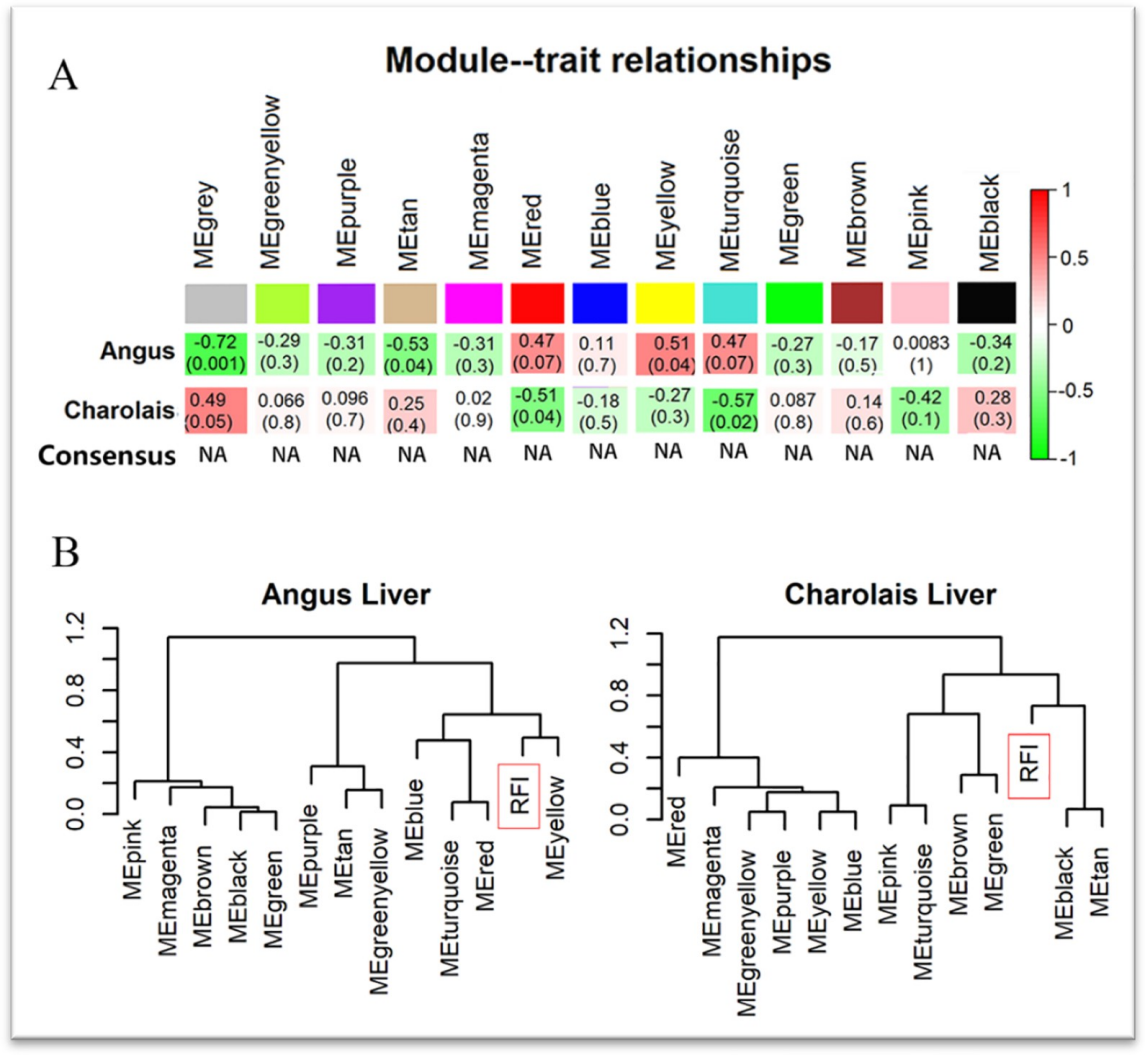
Construction of consensus network modules. **A**: Clustering diagram of Angus and Charolais cattle associated with RFI. **B**: Soft threshold was calculated based on network topology and connectivity, and the best soft threshold β of 8 was selected with R2 of 0.85. **C**: One-step method to construct the co-expression network, a branch from top to bottom represents a gene and points to the module below. **D**: Angus-specific and Angus-Charolais consensus module Fisher’s overlap test, where the numbers on the horizontal and vertical axis represent the number of genes within the module, the numbers in the figure represent the Angus-specific and consensus network gene overlap, the color represents significance, the darker the red, the more significant (the color represents the value calculated as -1*log(p)).

Prior to analysis, the samples were clustered using the hclust function in the R package WGCNA. Overall, the clustering results indicated high intra-group similarity and low inter-group correlation (Euclidean distance) in the sample (Fig 2A). When the scale-free topology model fit reached 0.85 (R = 0.85), a soft threshold β = 8 was assigned to construct the Angus-Charolais liver consensus module (Fig 2B). The results of consensus module showed that the vast majority of genes were grouped into related modules, especially the blue and brown modules (Fig 2C). A total of 17 RFI-related modules were generated by WGCNA analysis, of which turquoise, blue and brown contained the largest number of genes, 1,024, 1,097 and 798, respectively (Fig 2D, indicated by the vertical axis labels). Subsequently, the hypergeometric test was applied to investigate the overlap of RFI-related modules in Angus and Charolais livers, and it was found that many genes in several Angus-specific and consensus network modules had a high overlap, and this overlap reached a significant level (*P*<0.05). Meanwhile, this study found that the consensus module (Fig 2D), the horizontal axis labels show that there is less gene overlap between the consensus module (Fig 2D) and the counterpart modules, and the difference was not significant, indicating that the gene expression patterns of bovine liver tissues at the same RFI level are dramatically different, although the executive functions have little difference.

**Fig 3:**
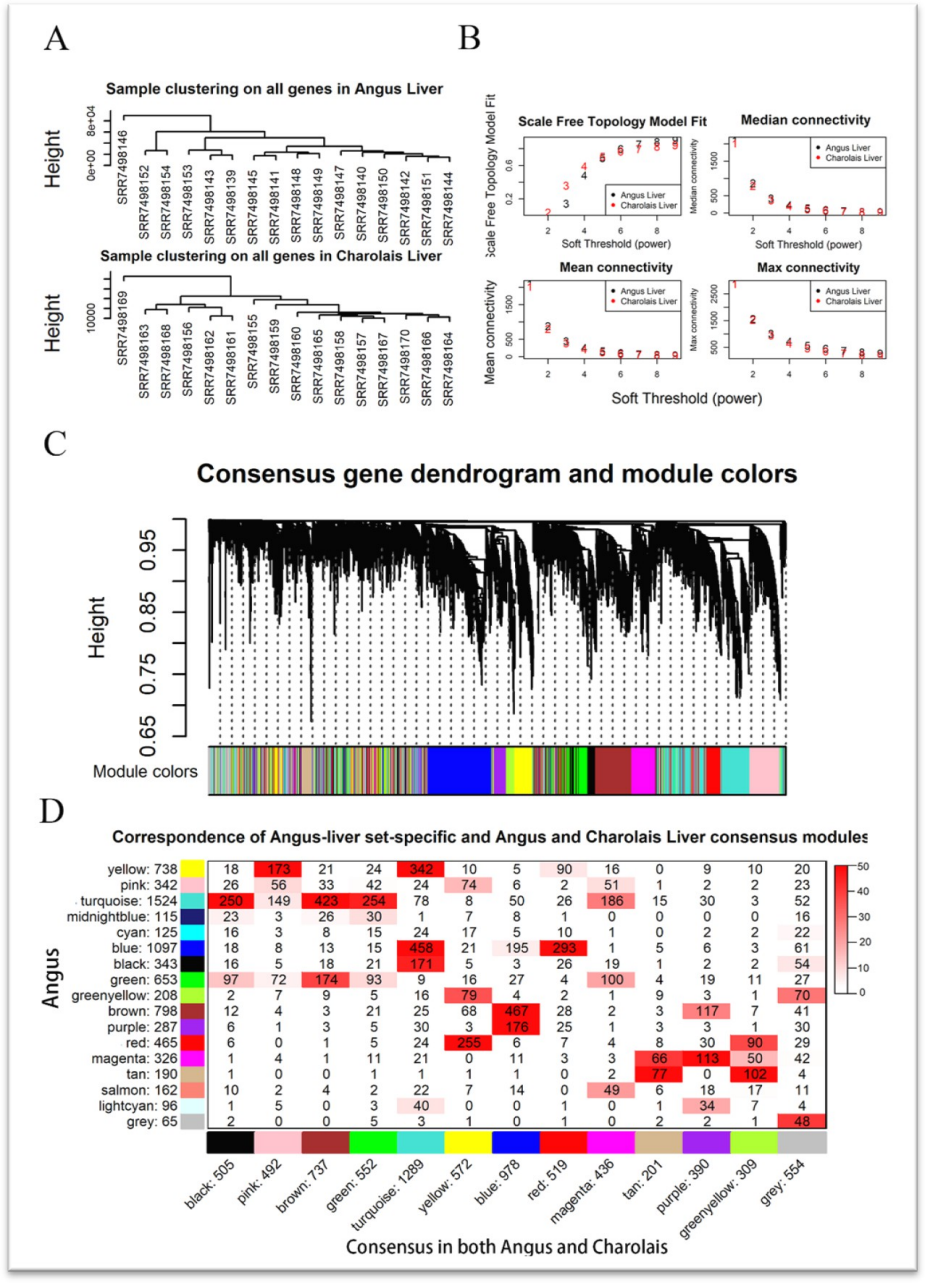
Identification of consensus modules for Angus and Charolais cattle liver tissues. **A**: Identification of consensus modules for Angus and Charolais cattle, where the row names "Angus" and "Charolais" represent the highly significant correlations between Angus and Charolais cattle liver gene expression modules and RFI, respectively, with darker colors representing stronger correlations, red representing positive correlations and green representing negative correlations. The row labelled "Consensus" is the consensus module analysis, calculated as follows: If the correlation coefficients used for species-specific and RFI have the same sign (zero relationships when the two correlations have opposite signs, labelled "NA"), the minimum correlation coefficient and the maximum significance test p-value were retained to assess the relationship between the two modules. **B**: Correlation analysis of Angus and Charolais RFI with the corresponding modules; the closer the branches are, the stronger the correlation.

Once the modules were identified, the common modules between Angus and Charolais cattle were taken for analysis (Fig 3). In Angus cattle, the relevant modules were (P < 0.07 and r > 0.4, ordered by the absolute value of the correlation coefficient) grey, tan, yellow, red and turquoise modules. In Charolais cattle they were turquoise, red and grey (the grey module gene was the gene not assigned to the specified module (Fig 3A); the correlation between the same module in Angus and Charolais cattle was reversed and marked "NA", indicating that gene expression in liver tissues was more variable at the same level. In Angus liver (Fig 3B, left), the most highly correlated modules with RFI were yellow (r = 0.51, *P* = 0.04), red (r = 0.47, *P* = 0.07), turquoise (r = 0.47, *P* = 0.07) and tan (r = -0.53, *P* = 0.04); in Charolais liver (Fig 3B, right), red (r = -0.51, *P* = 0.04), turquoise (r = -0.57, *P* = 0.02). Among these modules, the turquoise and red modules showed a significant negative correlation in Angus and Charolais, which could be one of the main clues to uncover the association of different beef breeds with RFI.

### Functional enrichment analysis of key modules

**Fig 4:**
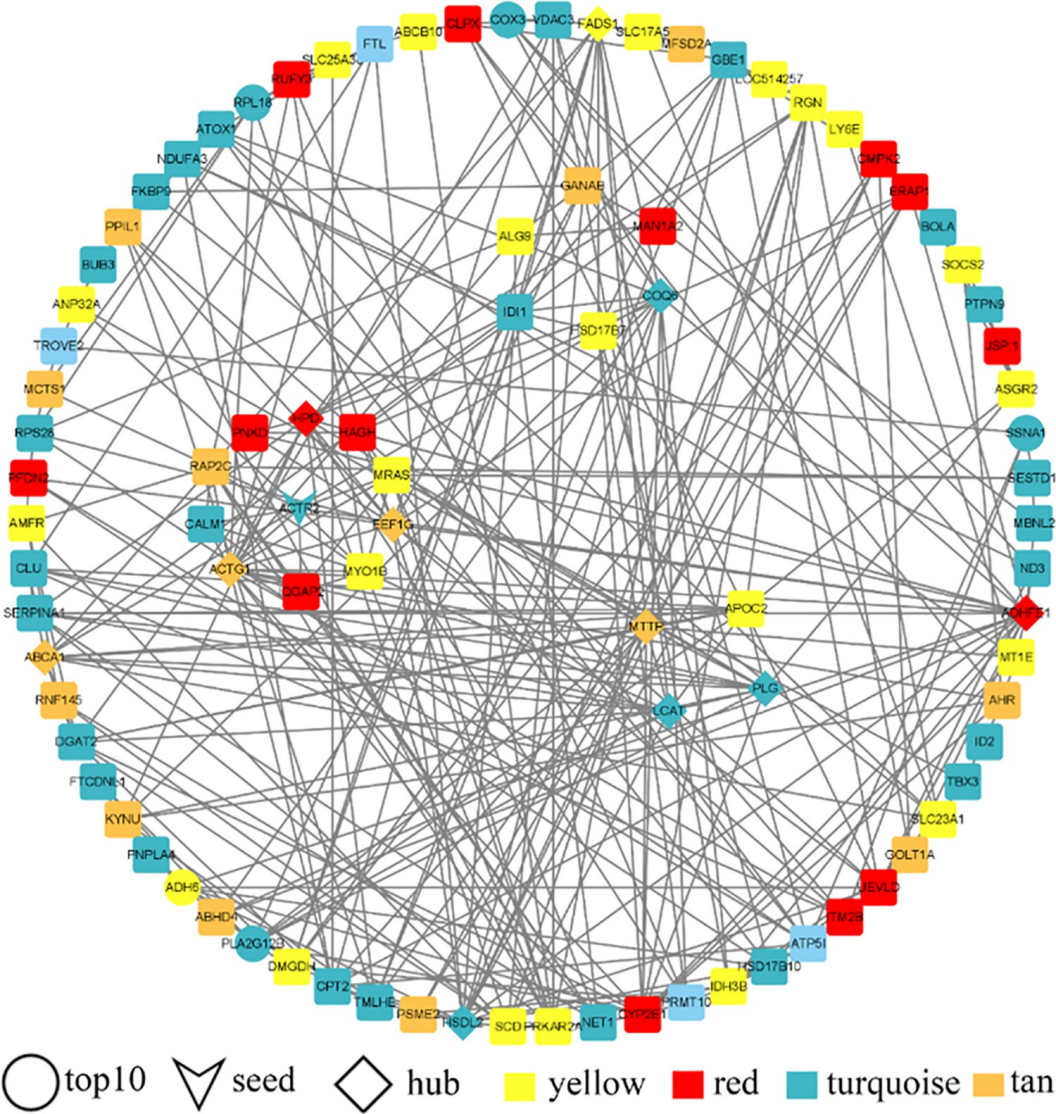
Enrichment analysis of Angus and Charolais cattle with significant negative correlation module (red module). **A**: Treemap plot of enrichment analysis results, box size represents the number of enriched genes, color represents the enrichment significance q-value. **B**: Treeplot plot of enrichment analysis results, the tree on the left represents the cluster analysis according to BP, the background color is the same to represent; the background color is the same as the function of a class, e.g. brown color represents "carboxylic organic molecule", others are the later description; the color of the graph note indicates the enrichment significance. On the right is the heatmap of the enrichment pathway of the genes *ADHFE1*, *HAGH*, *HPD* and *PNKD*. The color represents the Z-value of GS (Z.GS.meta) from the meta-analysis of Angus and Charolais cattle.

In the key module identification section, the turquoise and red modules were found to be significantly negatively correlated in Angus and Charolais. Following the meta-analysis of the red module in Angus and Charolais cattle, further enrichment analysis was performed for genes with absolute Z-score values greater than or equal to 2 (29 genes in total, S3 Table). The results showed that under threshold conditions (*q-value* < 0.05), these genes were only enriched in biological processes related to biological processes (Fig 4A) and mainly related to metabolic processes, such as ’small molecule catabolic process’, ’organic acid metabolic process’ and ’carboxylic acid metabolic process’. The analysis of these biological processes showed that they were mainly classified as ’carboxylic acid organic molecule’ related (Fig 4B), and the genes involved were mainly *ADHFE1*, *HAGH*, *HPD*, *PNKD*, indicating the different metabolism of organic compounds in the liver. These results suggest that differences in the metabolism of organic compounds in the liver may be the main factor in the variation of RFI in beef cattle of various breeds.

**Fig 5:**
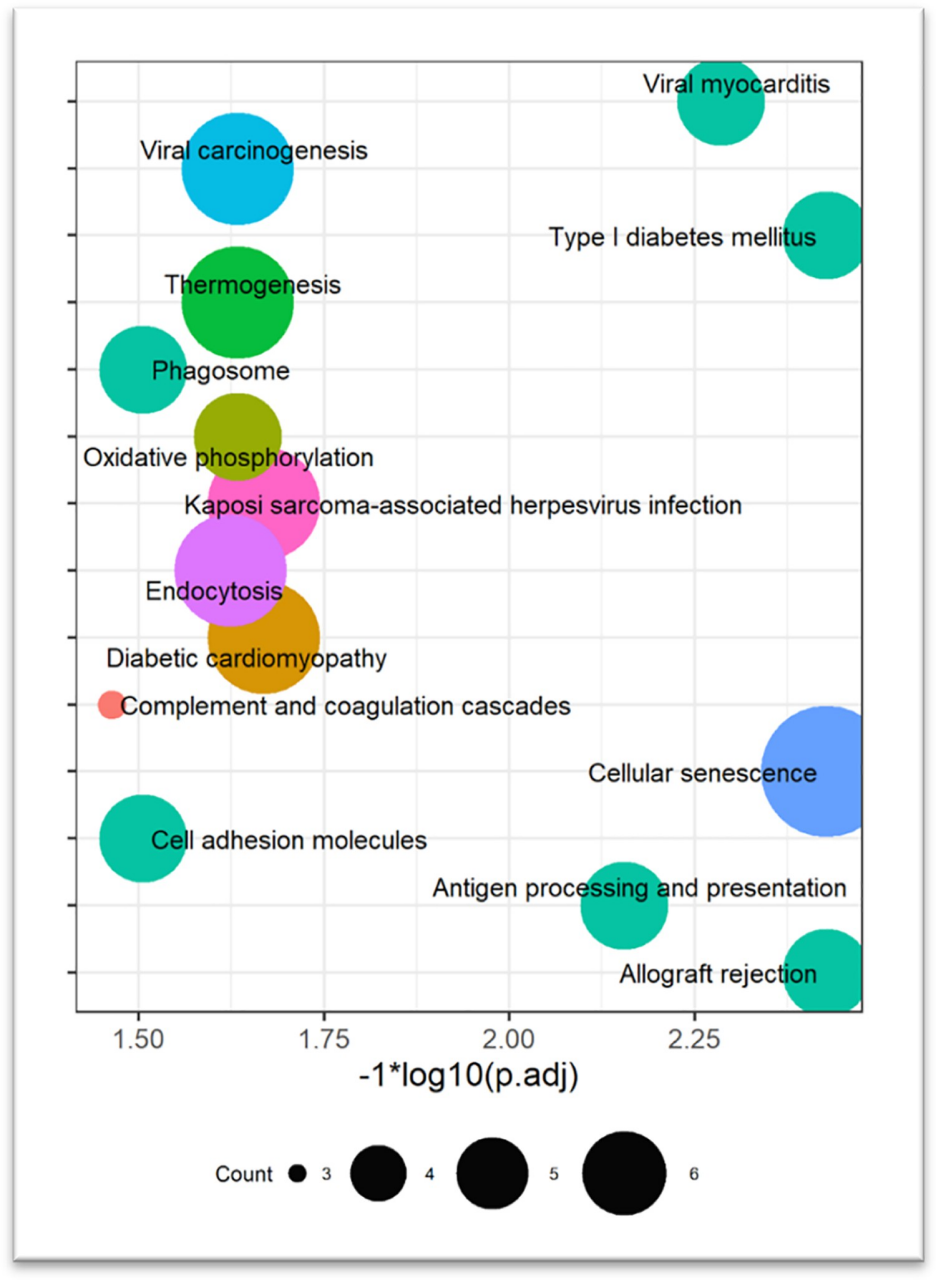
Functional enrichment analysis of the significantly negatively correlated turquoise module in Angus and Charolais cattle. The size of the circles represents the number of genes contained in the pathway; the color has no practical significance. The horizontal axis is "${ln(padj)}^{-1}$", which is 1.30 when *padj* = 0.05. The larger the *padj*, the larger the ${ln(padj)}^{-1}$.

In the turquoise module (containing a total of 78 genes satisfying the condition, | Z-Score | ≥ 2, S3 Table), genes were enriched in KEGG signaling pathways (Fig 5), such as the energy metabolism-related signaling pathway "oxidative phosphorylation", "thermogenesis", and the immune response/disease-related signaling pathway "antigen processing and presentation", "cellular genesis". "thermogenesis"; immune response/disease-related signaling pathway "Antigen processing and presentation", and "cell adhesion molecules", "diabetic cardiomyopathy"; and cellular activity-related signaling pathways "cellular senescence" and "endocytosis".

**Fig 6:**
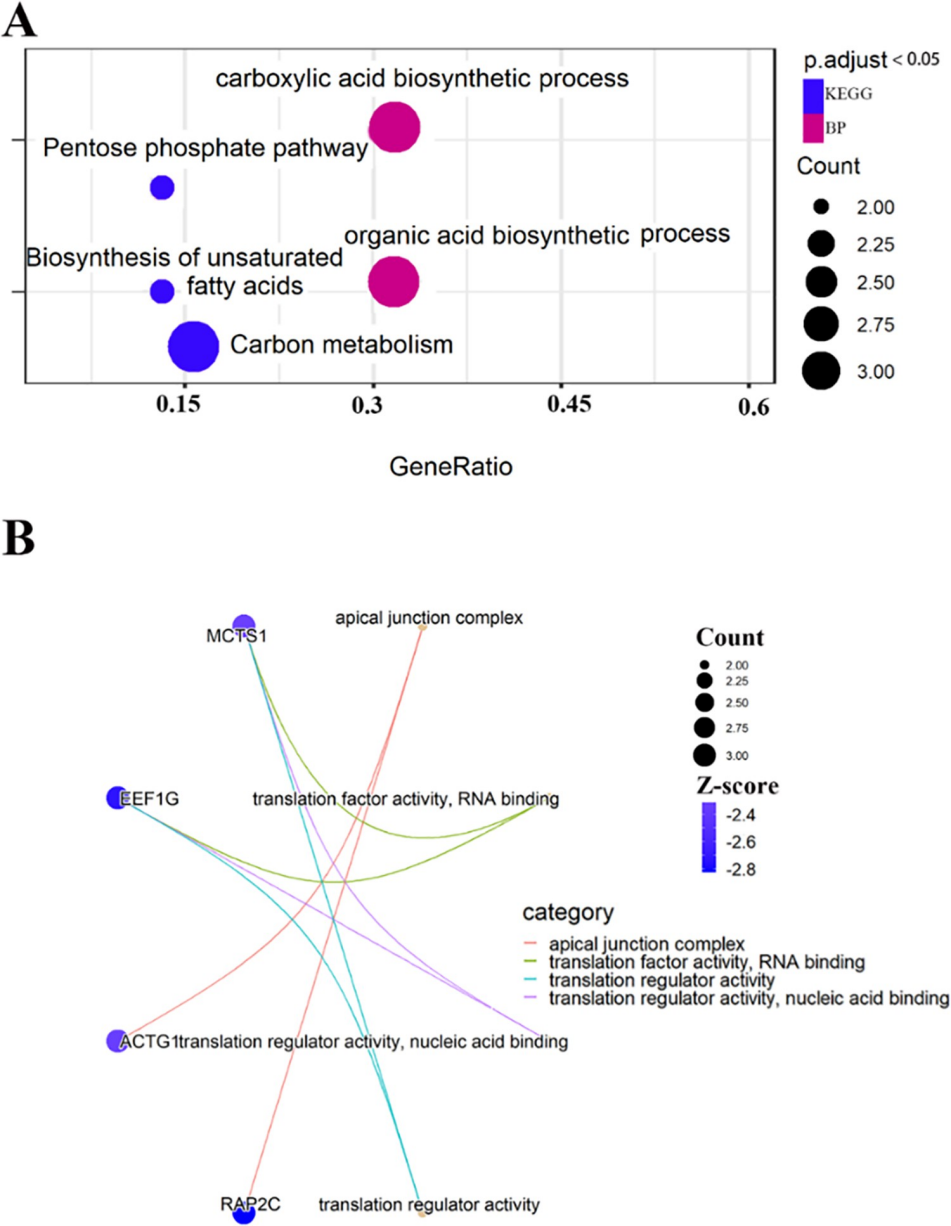
Functional enrichment analysis of the brown and yellow modules of Angus and Charolais cattle with significant negative correlation. **A**: Dotplot of functional enrichment analysis and pathway analysis results of the yellow module, where the color indicates the functional classification, blue is the KEGG pathway, pink is BP, and the size of the circle represents the number of enriched genes under the term. **B**: Functional enrichment analysis and pathway analysis results of the tan module cnetplot, where the color intensity represents the size of the Z-score value, the size of the circle represents the number of enriched genes in the term, and the blue dot represents the specific gene name.

Among the yellow and brown modules, which were significant in Angus and not in Charolais, the yellow module (containing a total of 30 genes fulfilling the condition, | Z-score | ≥ 2, S3 Table) was mainly enriched in metabolic pathways (Fig 6A), such as ’pentose phosphate pathway’, ’unsaturated fatty acid biosynthesis’, ’carbon metabolism’; for functional enrichment, they are mainly in organic acid metabolic processes, such as ’carboxylic acid biosynthetic process’ and ’organic acid biosynthetic process’. In the tan module (containing a total of 18 genes that meet the conditions, | Z score | ≥ 2, S3 Table), genes mainly perform translation-related functions (Fig 6B), such as ’translation factor activity, RNA binding’, ’translation regulator activity’ and ’translation regulator activity, nucleic acid binding’. These results suggest that biological processes related to substance metabolism and protein translation in Angus liver may be one of the factors causing the variation in RFI in well-bred beef cattle.

### Identification of key genes

**Fig 7:**
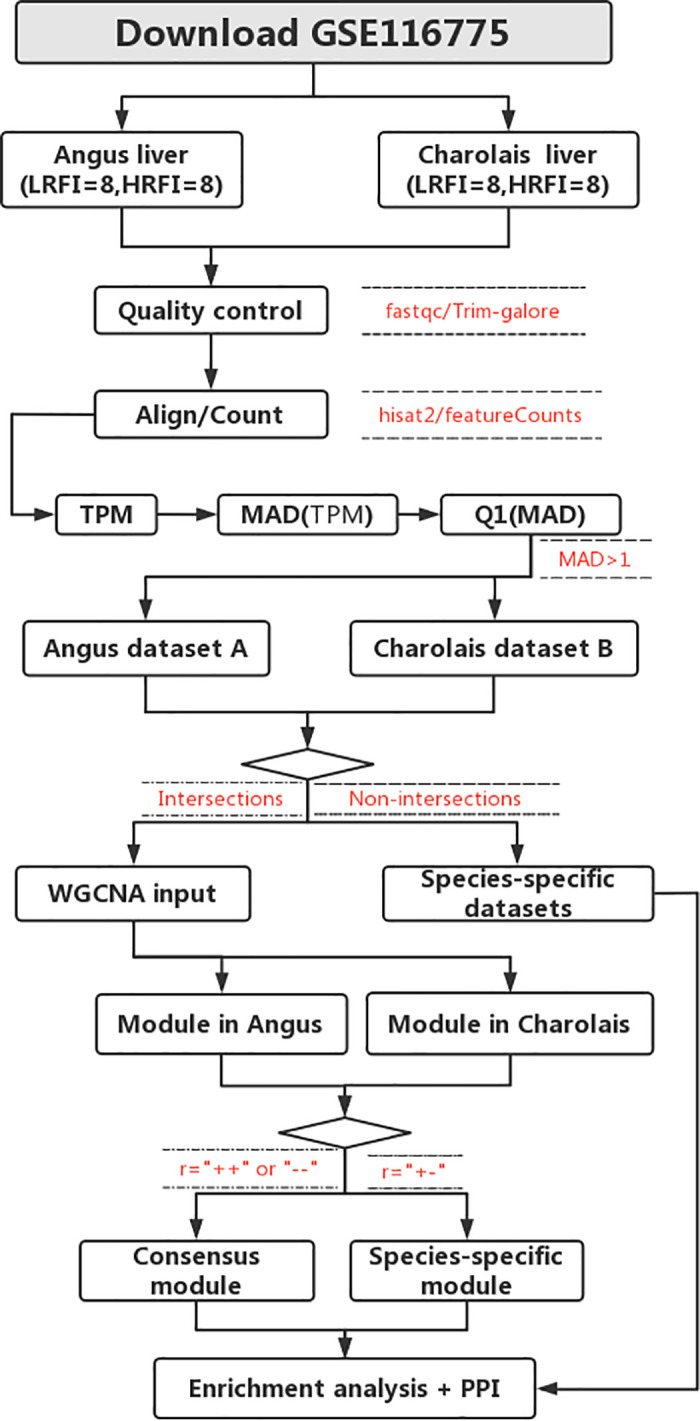
PPI analysis of genes selected from modules with | Z score | ≥ 2 genes. These genes are from the yellow module (30 genes, fill colour is yellow), red module (29 genes, fill colour is red), tan module (18 genes, fill colour is tan) and turquoise module (78 genes, fill colour is turquoise). The ’circle’ shape represents the top 5 genes when | Z-Score | > 0, with a total of 10 genes, and is labelled ’Top10’. The shape "V" represents the seed genes used to build the PPI network genes when the core subnetwork is calculated by the Cytoscape plugin MCODE, and is labelled "seed". The ’diamond’ shape represents the gene used to build the PPI network gene by the Cytoscape plugin Cytohubba to calculate the Degree, EPC, MCC and MNC values of each gene and retain the intersection of the top 20 genes for each gene to obtain a total of 11 hub genes, labelled ’hub’. The ’square’ shape represents the other genes used to construct the PPI network. The large outer circle represents the non-core subnetwork genes and the three modules inside the circle (from most to least number of genes) are the three core subnetworks with scores of 5.6, 4.5 and 2.8 respectively.

PPI analysis was performed to further elucidate the significant correlation modules turquoise, red, yellow and tan modules in | Z-score | ≥ 2 gene correlations and to identify key genes. The results showed that the network contained a total of 91 nodes and 323 edges. MCODE plugin was used to analyze the network and obtained three core subnetworks (3 modules in the circle in Fig 7), among which there is a core subnetwork with a score > 5 (the one with the highest number of genes), whose seed gene is the actin-related protein two gene (*ACTR2*, the gene with the "V" shape in Fig 7). Meanwhile, 11 core genes were detected (the genes with the shape of "diamond" in Fig 7), namely *ABCA1*, *ACTG1*, *ADHFE1*, *COQ6*, *EEF1G*, *FADS1*, *HPD*, *HSDL2*, *LCAT*, *MTTP* and *PLG*. In addition, the top 5 genes with the highest (positive) and lowest (negative) Z scores were included in the core candidate gene pool, namely *ADH6*, *BCL7C*, *C3H1orf50*, *COX3*, *EMC9*, *PLA2G12B*, *RAMP2*, *RNF19A*, *RPL18*, *SSNA1*. In summary, 22 key candidate genes were identified (Table 1).

**Table 1:** List of key genes.

GeneSymbol	Description	Category	Source
*ADHFE1*	alcohol dehydrogenase iron containing 1	hub	red
*BCL7C*	BAF chromatin remodeling complex subunit	top10	red
*HPD*	4-hydroxyphenylpyruvate dioxygenase	hub	red
*ABCA1*	ATP binding cassette subfamily A member 1	hub	tan
*ACTG1*	actin gamma 1	hub	tan
*EEF1G*	eukaryotic translation elongation factor 1 gamma	hub	tan
*MTTP*	microsomal triglyceride transfer protein	hub	tan
*ACTR2*	actin related protein 2	seed	turquoise
*C3H1orf50*	chromosome 3 C1orf50 homolog	top10	turquoise
*COQ6*	coenzyme Q6, monooxygenase	hub	turquoise
*COX3*	cytochrome c oxidase subunit III	top10	turquoise
*EMC9*	ER membrane protein complex subunit 9	top10	turquoise
*HSDL2*	hydroxysteroid dehydrogenase like 2	hub	turquoise
*LCAT*	lecithin-cholesterol acyltransferase	hub	turquoise
*PLA2G12B*	phospholipase A2 group XIIB	top10	turquoise
*PLG*	plasminogen	hub	turquoise
*RAMP2*	receptor activity modifying protein 2	top10	turquoise
*RNF19A*	ring finger protein 19A, RBR E3 ubiquitin protein ligase	top10	turquoise
*RPL18*	ribosomal protein L18	top10	turquoise
*SSNA1*	SS nuclear autoantigen 1	top10	turquoise
*ADH6*	alcohol dehydrogenase 6 (class V)	top10	yellow
*FADS1*	fatty acid desaturase 1	hub	yellow

## Discussion

RFI is a complex quantitative trait controlled by polygenes [30], and it determines the development of the beef cattle industry. As an important economic trait, researchers have tried to uncover the signaling molecules associated with the trait in different tissues such as liver [14], skeletal muscle [15], blood [16], adipose tissue [17], rumen epithelium [18], duodenum [2]. However, the characteristics and commonalities of RFI in different breeds of beef cattle are not well understood. The liver is the central organ of systemic metabolism and plays an essential role in maintaining lipid homeostasis [31]. In the present study, Angus and Charolais livers were selected for transcriptome sequencing to investigate the characteristics and commonalities.

As expected, several significantly correlated modules were identified in Angus and Charolais cattle. The yellow, red and turquoise modules were found to be significantly positively correlated with RFI in Angus cattle, whereas the last two modules were significantly negatively correlated in Charolais cattle. In our previous study [13], the magenta (positive correlation) and pink (negative correlation) modules were found to be significantly correlated in the liver of Charolais cattle with different diet compositions. In a study by Salleh et al [32], more significantly correlated modules were reported in the liver of RFI cattle (Holstein, Jersey) with different diet compositions (12 for Holstein and 4 for Jersey). In our study, these transcriptomic data came from the same experimental conditions and the same modules were correlated with RFI levels, which were consistent between Angus and Charolais cattle. These findings are similar to those described above: different beef breeds and rearing and management methods could be sources of variation. Therefore, different batches, species, physiological stages and feeding management may lead to considerable variation between the liver transcriptomes of beef cattle with the same level of RFI.

In the red and turquoise module, genes were mainly enriched in the corresponding signalling pathways of nutrient and energy metabolism, suggesting that nutrient and energy metabolism may be the main reason for the differences in feed efficiency between Angus and Charolais cattle. FE has been found to be closely related to substance and energy metabolism, such as the high FE animals in cattle rumen [18] and skeletal muscle [33], chicken skeletal muscle [33], and pig skeletal muscle and liver [34,35] also showed upregulation of genes associated with the electron respiratory chain. The electron respiratory chain is the primary site of energy production in the organism and can generate significant amounts of ATP. Therefore, the overall efficiency of the electron respiratory chain may be a potential determinant of FE. It has also been shown that mitochondrial respiration rates are higher in individuals with high FE than in those with high RFI [36], and that mtDNA copy numbers are significantly lower, indicating lower electron transport chain uncoupling and oxidative stress [37,38]. In addition, the expression of genes involved in the tricarboxylic acid cycle and oxidative phosphorylation was relatively high in skeletal muscle and liver [36,39], and ATP synthesis was more efficient [40–42]. Therefore, high FE organisms may have a more optimal layout of the electron respiratory chain or more efficient substance metabolism, with less mitochondrial uncoupling, stress response and mitochondrial number, which improves energy efficiency. In parallel, genes in the turquoise module have been found to be involved in the body’s immune function and disease-related pathways. Studies in different species and tissues have shown that the upregulated genes in low FE chicken breast muscle were mainly involved in immune and inflammatory responses [41]; the highest differential expression enrichment in pig liver were those involved in protein ubiquitination, followed by immunomodulation related pathways [43]. In the Angus cattle skeletal muscle transcriptome, differential genes were significantly enriched in the immune and inflammatory response [33]. The immune and inflammatory response is highly energy intensive, resulting in fewer nutrients available for its production [44]. At the same time, it has been suggested that animals with high FE have a more efficient ability to resist inflammation and devote more energy to growth and muscle deposition [45]. It is worth noting that of the significantly related modules yellow and tan, which are unique to Angus cattle, the former is mainly involved in signalling pathways related to glucolipid metabolism, whereas the latter is involved in protein translation processes, suggesting that hepatic metabolism and energy metabolism are more active or efficient in Angus cattle than in Charolais cattle. In addition, we found similar results to Mukiibi’s study [46], mainly involving fatty acid, carbohydrate and energy metabolism. Meanwhile, they also found that low RFI cattle reduced liver fat synthesis and accumulation, thus improving feed efficiency. These results suggest that the genes associated with RFI between Angus and Charolais are mainly involved in immune and metabolic aspects of the organism and that these processes may be the main reason for the opposite correlation of the same module in Angus and Charolais cattle.

A group of key genes closely related to fatty acid metabolism were identified: *PLA2G12B*, *MTTP*, *LCAT*, *ABCA1* and *FADS1*. Liver expression of *MTTP* genes is associated with lipid metabolism [47]. *MTTP* is involved in the regulation of lipid homeostasis and serum lipid levels [48], protection against cellular stress caused by lipid overload [49], and deletion of this gene results in an inability to secrete apolipoproteins [49], very low and low density lipoprotein triglyceride levels [50]. *ABCA1* gene expression is closely associated with cholesterol efflux [51], and its primary function is to promote cellular cholesterol and phospholipid efflux [52], and inhibition or knockdown of expression can lead to intracellular lipid accumulation [53,54]. Knockdown of *MTTP* and *ABCA1* in the mouse intestine significantly reduces plasma cholesterol concentrations and increases intestinal triglyceride and free fatty acid concentrations [55]. The *LCAT* gene plays a central regulatory role in HDL metabolism and reverses cholesterol transport, improving lipid metabolism and esterification of free cholesterol [56]. The *LCAT* knockout mice are resistant to obesity, hepatic endoplasmic reticulum stress and hepatic endoplasmic reticulum cholesterol [57]. It has also been shown that LCAT gene expression is upregulated in the liver of pigs with low RFI [58]. The *PLA2G12B* gene controls triglyceride metabolism. When this gene is deficient in mice, hepatic and LDL triglyceride secretion is impaired [59], and liver and plasma triglyceride and fatty acid concentrations are significantly reduced [60], which in turn is involved in the regulation of hepatic very-low-density lipoprotein triglycerides. The *FADS1* gene can affect plasma levels of polyunsaturated fatty acids, such as arachidonic/linoleic acid [61], and its polymorphisms are closely associated with LDL and triglycerides [62]. The liver is the lipid regulator in the organism. After the animal has been fed, fats are digested in the small intestine into triglycerides and absorbed into the bloodstream. The liver is responsible for about 20% of the triglycerides entering the blood, which undergo β- or ω-oxidation for energy in the liver mitochondria and peroxisomes. Meanwhile, the liver can perform glycolysis to produce dihydroxyacetone phosphate and acetyl coenzyme A to participate in fatty acid synthesis. Of the five key genes selected, *PLA2G12B*, *LCAT*, *MTTP*, *LCAT*, *ABCA1* and *FADS1*, the remaining three genes were derived from brown and yellow modules that were significantly associated with RFI in Angus cattle, except for the first two genes that were derived from turquoise, a significantly negatively associated module. Therefore, differences in liver fatty acid related metabolism and expression of key genes may be one of the reasons for the negative correlation of the same module in Angus and Charolais cattle.

## Conclusion

The livers of beef cattle with the same level of RFI perform similar functions, but the expression pattern of key genes varies greatly between breeds, which may allow large differences in the efficiency of carrying out biological processes such as nutrient and energy metabolism, resulting in different feed efficiency between breed of cattles. Although these results explain the potential reasons for the variation in RFI caused by different breeds of beef cattle, they still need to be proven by further functional experiments.

## Supporting information

S1 Table Profile of gene expression in Angus. (XLSX)

S2 Table Profile of gene expression in Charolais. (XLSX)

S3 Table Gene for significantly related modules. (XLSX)

## Abbreviations

FE: feed efficiency
PPI: protein-protein interaction
WGCNA: weighted correlation network
RFI: residual feed intake
TPM: transcripts per kilobase of exon model per million mapped reads
MAD: median absolute deviation
MM: module-memberships
KME: eigengene connectivity
EPC: edge percolated component
MCC: maximal clique centrality
MNC: maximum neighborhood component
BP: biological process
MF: molecular function
CC: cellular component
KEGG: Kyoto Encyclopedia of Genes and Genomes
